# Association Between Erosive Esophagitis and the Anthropometric Index in the General Korean Population

**DOI:** 10.4274/balkanmedj.galenos.2018.2018.0523

**Published:** 2019-05-10

**Authors:** Hyun Young Kim

**Affiliations:** 1 Department of Internal Medicine, Seoul National University Bundang Hospital, Seongnam-si, Korea

**Keywords:** Anthropometry, esophagitis, gastroesophageal reflux disease, koreans

## Abstract

**Background:**

An association between obesity and gastroesophageal reflux disease has been reported. However, previous studies have focused on obesity or central obesity.

**Aims:**

To investigate the association of the anthropometric index and endoscopic erosive esophagitis in health checkups of Koreans.

**Study Design:**

Case-control study.

**Methods:**

A total of 1.207 consecutive subjects (aged 40-80 years) during health checkups underwent upper endoscopy and bioelectrical impedance analysis. We collected anthropometric data by bioelectrical impedance analysis, which consisted of body mass index, percent body fat, muscle mass, and fat mass.

**Results:**

Of 1.207 subjects who underwent upper gastrointestinal endoscopy (mean age, 50.55±9 years), 239 (19.8%) had endoscopic erosive esophagitis. In a univariate analysis, the endoscopic erosive esophagitis group was more likely to be a male and had a higher body mass index, muscle mass and fat mass. In logistic regression analysis, only muscle mass remained an independent risk factor for EE after adjustment for both age and gender. Higher muscle mass was associated with increased EE risk (rate ratio: 1.354, 95% confidence interval: 1.206-1.405, p= 0.027).

**Conclusion:**

High muscle mass, but body mass index, is an independent risk factor for erosive esophagitis in a population over 40 years of age.

Gastroesophageal reflux disease (GERD) is caused by the reflux of gastric acid or food into the esophagus, which causes heartburn or reflux (1). GERD is classified into erosive esophagitis (EE) and non-erosive GERD. GERD can seriously impair quality of life and lead to Barrett’s esophagus when left untreated and can cause an esophageal ulcer, esophageal stricture, and tumor (1). In Western Europe and North America, the rate of people experiencing gastroesophageal reflux symptoms at least once a week is as high as 20% to 30% of the population (2). In Korea, the prevalence of GERD is 14.7% to 17.4%, and the prevalence of EE is 8.6% to 11.8%, which is lower than that of Western countries (3,4,5). However, recently, GERD is on the rise because of the increase in life expectancy, adoption of a Western-type diet, and obesity in Asian countries. The prevalence of GERD is increasing in Korea, and the prevalence of reflux esophagitis was 1.3% in the 1980s, 2.1% in the early 1990s, 5.4% in 1997, 7.0% in 1999, 8.0% in 2006, and 8.8% in 2011 (2,3,4,5).

The exact reason why the prevalence rate is increasing is unknown, but it is likely that Western eating habits and an increase in the obese population have been contributing factors. The mechanisms of GERD are varied and have been reported as lower esophageal sphincter function, abnormal esophageal motility, gastric hyperplasia, delayed gastric emptying and reduced resistance of esophageal mucosa. The most important pathogenesis of GERD is believed to result from the temporary relaxation of the lower esophageal sphincter. In addition, anatomical deficits such as a hiatal hernia, low pressure of the lower esophageal sphincter, and increased gastroesophageal pressure may increase gastroesophageal reflux. This reflux may be caused by various factors such as an increase in proximal pressure; however, it may also result when the amount of muscle is increased, the abdominal pressure is increased, and the proximal pressure of the gastroesophageal muscles is increased.

The aim of this study was to investigate risk factors related to EE in the Korean general population.

## MATERIALS AND METHODS

### Anthropometric data

The study population consisted of all gastroscopies and anthropometric data performed in a single tertiary hospital. The target population enrolled in this study consisted of mostly public office or company workers and families who are required to have a mandatory group screening program. This program includes tests that are not personally selectable and must be performed on a mandatory and annual basis, such as upper gastrointestinal endoscopy, anthropometric index, blood tests, abdominal ultrasound, electrocardiogram, and chest X-ray examinations.

The study group consisted of 1207 subjects aged 40 to 80 years who had undergone a complete screening upper gastrointestinal endoscopy for an average risk for gastric cancer. Anthropometric data included height, weight, body mass index (BMI), percent body fat, fat mass, and muscle mass. Weight was measured to the nearest 0.1 kg. Height was measured to the nearest 0.1 cm. Weight was measured using an Inbody S-10 (Inbody Co, Ltd, Seoul, Korea), and BMI was calculated as weight in kilograms divided by height in meters squared (kg/m^2^). All anthropometric results were based on a single-body measurement examination.

A single tertiary Hospital Health Promotion Center provided various screening packages of exams including upper gastrointestinal endoscopy. All screened subjects volunteered or were employer-sponsored to undergo upper gastrointestinal endoscopy regardless of age (even asymptomatic subjects in their 20s and 80s who were undergoing routine checkups). Study subjects with a history of gastrointestinal surgery or with a respiratory problem requiring current medication or pregnant subjects were excluded. This study was approved by the Institutional Review Board (IRB No. B-1708-412-105). This study had a retrospective design and written informed consent was waived by the Institutional Review Board (IRB No. B-1708-412-105).

### Endoscopy exam

Upper gastrointestinal endoscopy was conducted on all study subjects by five expert gastroenterologists (>10 years of endoscopic experience) certified by the Korean Society of Upper Gastrointestinal Endoscopy using conventional white light videoscopy (GIF-H260 or GIF-H290; Olympus, Aizu, Japan). The grade of EE seen on upper gastrointestinal endoscopy was classified from A to D according to the Los Angeles (LA) classification (6). All endoscopic images of EE were stored as pictures on the hospital network, namely the PACS system, and were reviewed by a single well-trained gastroenterologist. All gastroenterologists participated in the meeting who agreed to the consensus of EE findings.

### Statistical analysis

Data were analyzed using SPSS software (version 21.0, SPSS Inc., Chicago, IL, USA). Continuous variables are expressed as mean ± standard deviation whereas the categorical variables are expressed as absolute values and percentages. Medians and ranges are presented for continuous variables and percentages for categorical variables. Differences between variables were assessed by χ2 tests. All p-values were two-sided, and p<0.05 was considered statistically significant. The relationships between various variables were investigated using multiple regression analysis. The relationship among age, gender, BMI, percent body fat, muscle mass, fat mass, and endoscopic EE was assessed using a binomial logistic regression analysis model with these 6 parameters as independent variables.

### Bioelectrical impedance analysis

A multi-frequency bioelectrical impedance analyzer, InBody S5 Biospace device (Inbody Co, Ltd, Seoul, Korea/Model 720), was used according to the manufacturer’s guidelines. Bioelectrical impedance analysis estimates body composition using the difference in conductivity of various tissues given different biological characteristics of subjects. Conductivity is proportional to water content, and more specifically, to electrolytes, and it decreases as the cell approaches a perfect spherical shape. Adipose tissue is composed of round-shaped cells and contains relatively little water than other tissues such as muscle; therefore, conductivity will decrease as body fat increases. In practice, electrodes are placed at six precise tactile points of the body to achieve a multi-segmental frequency analysis. A total of 30 impedance measurements are obtained using six different frequencies (1, 5, 50, 250, 500, and 1000 kHz) at the five following segments of the body, namely right and left arms, trunk, and right and left legs.

## RESULTS

### Clinical characteristics of study subjects

This study included 1207 health checkup subjects aged 40 years or more (mean age: 55±9 years) for screening upper gastrointestinal endoscopy at a single tertiary Hospital Health Promotion Center. The mean age of subjects was 55 years (standard deviation ±9 years), and 62% were men. The prevalence of EE after 40 years of age was 19.8%. Barrett’s esophagus was not observed in this study.

Tables 1 and 2 show the differences in demographic and clinical characteristics between subjects with EE and those groups without EE. In the univariate analysis, the subjects with EE, when compared with those without EE, had a higher percentage of males (p<0.001), BMI (p<0.001), muscle mass (p<0.001), and fat mass (p<0.001). No significant difference in the prevalence of EE was found by age or percent body fat.

Male subjects were more numerous in the group with EE than in those without EE (180 vs 59, respectively, p<0.001). The BMI was higher in the group with EE when compared with that without EE (25.3±3.4 vs 23.9±3.1, respectively, p=0.000). Muscle mass was higher in the group with EE when compared with that without EE (48.8±8.7 vs 44.6±9.0, respectively, p<0.001), and so was fat mass (19.3±5.8 vs 17.7±6.1, respectively, (p<0.001).

Based on endoscopic exams, by LA classification, 239 subjects (19.8%) were found to have EE: 96 (40.2%) in LA-A, 127 (53.1%) in LA-B, and 16 (6.7%) in LA-C. The age and gender distributions of these 239 subjects with EE are shown in Table 3.

### Associations of endoscopic EE with components of anthropometric data

Results of multiple logistic regression analysis associations of EE and components of anthropometric data are shown in Table 4. Muscle mass remained an independent risk factor for EE after adjusting for both age and gender. Higher muscle mass was associated with an increasing risk for EE (odds ratio: 1.354, 95% confidence interval: 1.206-1.405, p=0.027). No significant interactions were found between endoscopic EE and the components of anthropometric data, such as BMI, percent body fat, and fat mass.

## DISCUSSION

Until now, most studies have reported obesity as a risk factor of GERD. We found a positive correlation between EE and each of the following individual factors by analyzing the components of anthropometric data: male gender, high BMI, high muscle mass, and high fat mass. However, multivariate analysis revealed that only high muscle mass was associated with EE. This study found no correlation between EE and BMI or fat mass.

In a previous study, multivariate logistic regression analysis revealed that BMI, central obesity, waist circumference, or visceral fat area/subcutaneous fat area ratio is associated with EE. Whether obesity promotes gastroesophageal reflux is still under debate. GERD is known to have three mechanisms, namely, transient lower esophageal sphincter relaxation, strain-induced increased intra-abdominal pressure, and deglutitive lower esophageal sphincter relaxation (7). Notably, the increase in intra-abdominal pressure because of obesity contributes to EE. Some studies have suggested that the risk of GERD or EE increases with increasing BMI (2,8,9). Many previous studies have used BMI as an index of obesity. However, BMI can reflect part of obesity, and it does not reflect all components of body composition. There are some reports that the correlation between EE and BMI is not positively correlated and considered to be influenced by various factors (8,10).

Another type of obesity, also known as abdominal or central obesity, promotes gastroesophageal reflux that may be related to increased intra-abdominal pressure. One recent study demonstrated that belt compression increased acid reflux following a meal (11). The mechanism is that having a waist belt on versus off causes a marked increase in gastroesophageal reflux, most evident after a meal. The effect of a belt was very close to the gastroesophageal junction. At the gastroesophageal junction, the pH of the lining of the distal esophagus that was normally lined by squamous mucosa became similar to that of the proximal stomach. Therefore, combined high-resolution pH and manometry system could examine the mechanism of increased esophageal acid reflux induced by the belt. These findings support the mechanism of the association between central obesity and GERD. In addition, Carlson and Hirano (12) examined the complex mechanisms of reflux during running by new technology simultaneous high-resolution manometry, and pH-impedance in 10 healthy subjects after a meal, including transient lower esophageal sphincter relaxation (transient lower esophageal sphincter relaxation, aka “belch reflex”) and transient formation of a hiatal hernia.

Early studies have shown a correlation between BMI and GERD based on stationary esophageal manometry and 24-h pH manometry (13,14,15). Obesity measured by BMI increases intra-gastric pressure, the same as intra-abdominal pressure. Furthermore, a rise in intra-abdominal pressure elevates lower esophageal sphincter pressure. Mid-term studies have shown a correlation between central obesity and GERD, classifying central obesity as an external factor, and tightening the belt and intra-abdominal fat as internal factors (16,17). Central obesity measured by abdominal visceral adipose tissue volume, but not BMI or waist circumference, was associated with EE. Recent studies have suggested that running or abdominal compression as a mechanical factor is correlated with GERD, and results of combined high-resolution pH measurement and manometry should be used (11,18).

In most clinical situations, direct measurement of the intra-abdominal pressure is impractical, so a surrogate measure of intra-gastric pressure was chosen. Several studies have evaluated that BMI, waist circumference, and waist belt compression have a positive relationship with intra-gastric pressure and the gastroesophageal pressure gradient (11,13,17,19).

Surprisingly, no information is available on the effect of muscle mass on intra-gastric or intra-abdominal pressure. We hypothesized that trunk or abdominal muscles might cause gastroesophageal reflux by increasing intra-gastric or intra-abdominal pressure. In the present study, high muscle mass was associated with the risk of endoscopic EE. Several possibilities have been formulated to explain how muscle mass can cause EE. These results suggest that high muscle mass may influence the intra-abdominal pressure and subsequent esophageal acid exposure. We were able to predict that a significant correlation with abdominal muscle alone explains a relatively small part of the variance in mucosal breaks of the gastroesophageal junction. The role of central muscle mass points to a mechanical rather than hormonal mechanism that influences mucosal breaks of the gastroesophageal junction. Our study shows the importance of mechanical factors in the association between muscle mass and reflux esophagitis.

The current study has several strengths. It is the first study to investigate anthropometric data including BMI, percent body fat, muscle mass, and fat mass for endoscopic EE. In addition, all gastroscopies were performed by highly experienced endoscopists who obtained high-quality data. All endoscopic images were reviewed by one expert endoscopist for assessment of LA classification grades thereby minimizing the intra-observer variation. To complement the reproducibility of the anthropometric data, one examiner performed two consecutive bioelectrical impedance analysis measurements.

However, this study has some limitations. First, this study uses bioelectrical impedance analysis, not computed tomography, to measure anthropometric data. The accuracy of bioelectrical impedance analysis remains a concern (20,21). Second, we did not evaluate lifestyle factors, such as alcohol or smoking status.

In conclusion, the anthropometric index, exceptionally high muscle mass, was positively correlated with endoscopic EE. Our findings might explain the mechanism by which non-obese patients developed EE.

## Figures and Tables

**Table 1:**
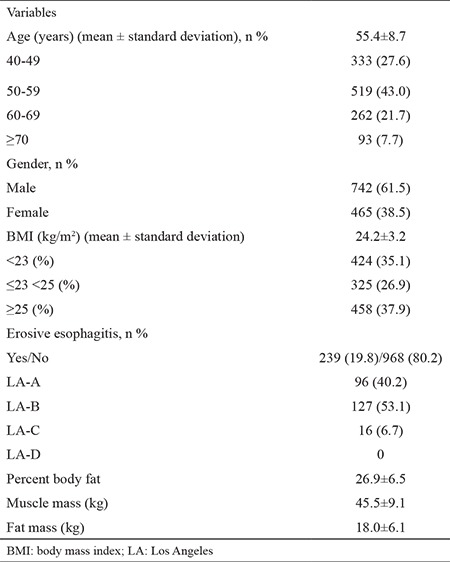
Demographics and baseline characteristics of subjects (n=1207)

**Table 2:**
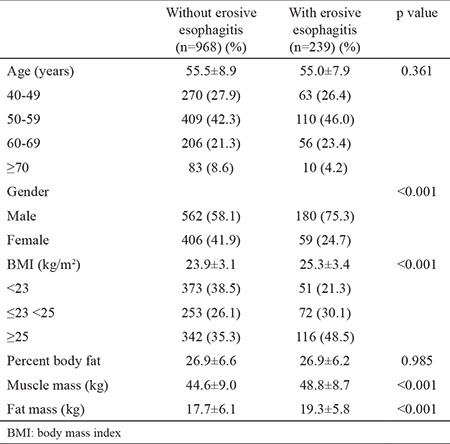
Comparison of characteristics between subjects with endoscopic erosive esophagitis and those without

**Table 3:**
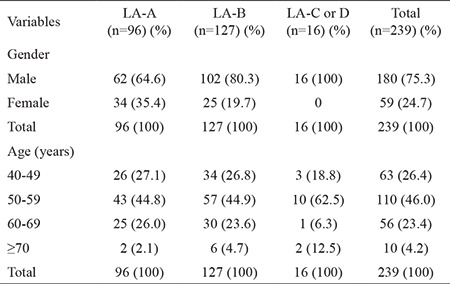
Erosive esophagitis characteristics according to the Los Angeles classification by age and gender

**Table 4:**
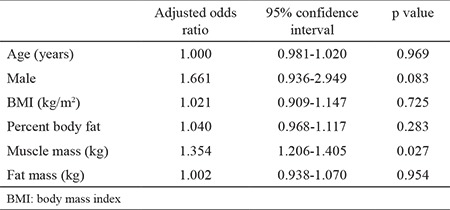
Logistic regression analysis of covariates for erosive esophagitis associations of erosive esophagitis and components of anthropometric data
